# Low-Cost Source Measure Unit (SMU) to Characterize Sensors Built on Graphene-Channel Field-Effect Transistors

**DOI:** 10.3390/s24123841

**Published:** 2024-06-14

**Authors:** Ashley Morgan Galanti, Mark A. Haidekker

**Affiliations:** School of Chemical, Materials and Biomedical Engineering, College of Engineering, University of Georgia, Athens, GA 30602, USA; amg19523@uga.edu

**Keywords:** source meter unit (SMU), graphene-channel field-effect transistor (G-FET), light sensing

## Abstract

This study introduces a flexible and low-cost solution for a source measure unit (SMU), which is presented as an alternative to conventional source meter units and a blueprint for sensor FET drivers. An SMU collects current–voltage (I-V) curves with an additional variable voltage or current and is commonly used to characterize semiconductors. We present the hardware design, interfacing, and test results of our SMU. Specifically, we present representative I-V curve measurements for graphene-channel FETs to demonstrate the SMU’s capability to efficiently characterize these devices with minimal noise and sufficient accuracy. This cost-effective solution presents a promising avenue for researchers and developers seeking reliable tools for sensor development and characterization. We demonstrate, with the example of surface illumination, how the sensing behavior of graphene-channel FETs can be characterized without the need for expensive equipment. Additionally, the SMU was validated with known passive and active components, along with probe station integration for semiconductor die-scale connection. The SMU’s focus on collecting I-V curves, coupled with its ability to identify device defects, such as parasitic Schottky junctions or a failed oxide, contributes to its utility in quality testing for semiconductor devices. Its low-cost nature makes it accessible for various research endeavors, enabling efficient data collection and analysis for graphene-based and other nanomaterial-based sensor applications.

## 1. Introduction

Nanomaterial-based field-effect transistor (FET) technology is a growing research area because of its broad sensing ability through the use of semiconducting nanostructures and surface recognition elements for selective detection of target analytes. Analytes can range from photons all the way to clinical biomarkers, and the nanomaterial used in the active gate region may include silicon nanowires, transition metal dichalcogenides, or carbon-based materials, such as graphene [1]. Graphene, used in this study as a representative example, is a two-dimensional, single-carbon-atom-layer material found as a hexagonal lattice known for its high electrical properties and thermal conductivity, along with optical and chemical properties that make them suitable as a sensor in a wide range of applications [2,3,4,5]. Microchips that use graphene are influenced by the gate potential with higher electron mobility, but also by environmental factors, such as photons and chemical reactants. Graphene-channel FETs have also been demonstrated to have good device stability, high carrier density, large surface-to-volume ratio, low-power and high-speed operation, and low-cost fabrication [6,7,8].

**Figure 1:**
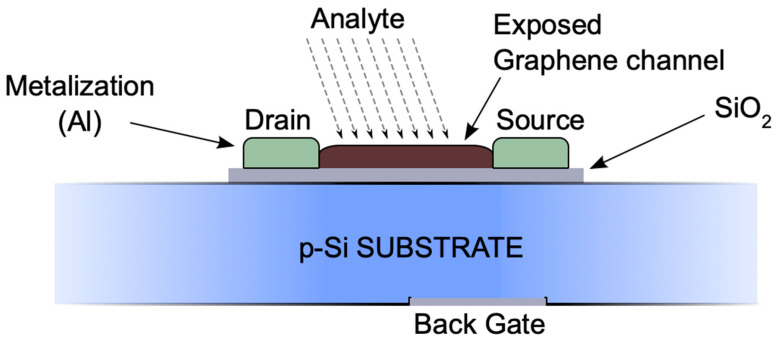
Schematic cross-section of a graphene-based sensor FET. The drain-source channel is formed of a graphene nanolayer, whose conductance is influenced by the gate electric field. The entire bulk silicon forms the gate, and a back-gate connection allows the gate potential to be applied. When the graphene is functionalized and binds to the target analyte, the conductivity changes (specifically, the Dirac point shifts), which, in turn, allows the detection of the analyte.

Graphene-channel FETs are used for a variety of gas, environmental, and biosensing applications, such as liquid-biopsy detection of exosomes for early cancer diagnosis [9], nitrate detection in water for quality monitoring [7], and virus detection of SARS-CoV-2 [8], to name a few examples. Typically, these devices monitor the shift of the Dirac point as a result of molecular binding on the sensor surface with the assistance of receptors that bind specifically to the detection target [9]. Other changes on the surface can cause a Dirac point shift, such as light source variation. As a result of graphene’s optical transparency, allowing up to 97.7% of light transmission in the visible range [10], its use in optical sensing applications is prominent. Graphene-channel FETs can detect and measure photon flux, converting light intensity into electrical signals. When exposed to incident photons, the device shows increased conductivity owing to photoinduced excitons and results in a shift of the electric field acting on the graphene. This shift changes the graphene’s conductivity, reflected in a changed characteristic relationship between the voltage across the sensor channel, $V_{DS}$, and the drain current, $I_{D}$ [8]. Applications of graphene-channel FET photodetection include high-speed communications, ultra-sensitive photodetectors, and radiation sensors, along with sensing applications for wearable electronics [11,12,13]. (See Figure 1).

The typical sensing application, therefore, aims to find the Dirac point by measuring the drain current $I_{D}$ as a function of the drain-source voltage $V_{DS}$ and with varying gate potential $V_{G}$. A shift of the Dirac point indicates the presence of the analyte; however, graphene FETs may face sensing challenges due to the highly sensitive gate channel and difficult-to-control properties of graphene, which can cause nontargets to be detected, add elevated noise levels, or prevent device functionality altogether. Another challenge is the Debye screening problem as the response of the graphene-channel FET is strongly dependent on phenomena occurring at the nanoscale interface paired with the additional complexity of the target sample and detection molecules and the low on/off ratio [14]. Hence, there is a need for specialized testing instruments. To fully characterize a graphene-channel FET, the basic $I_{D}\;$ over $V_{DS}$ curve needs to be acquired and repeated across different gate voltage levels. Here, $I_{D}$ needs to be acquired with sub-microampere precision while the gate voltage is varied over tens of volts simultaneously [15].

To address these measurement challenges, we developed a specialized device for acquiring the FET characteristic curves of a graphene-channel FET and other passive and active components containing up to three terminals. This device, referred to as a source measure unit (SMU), is an instrument that can supply a defined voltage or a defined current while simultaneously measuring voltage and current; in other words, it combines a sourcing and measurement function on the same pin or connector. Such SMUs are available commercially, but at costs that typically range from USD 5000 to USD 30,000 for options down to the sub-μA current range, as seen in Table 1. We introduce the hardware, primarily composed of analog components for voltage and current measurement and control, along with the software used for a low-cost, low-current SMU. Material costs are below USD 400, although a separate probe station must be available when the FET sensors need to be probed at the die level. The interface side of our hardware is flexible enough to be adapted to a variety of digital-to-analog converter (DAC) cards or dataloggers. We demonstrate the measurement capabilities of our SMU in a variety of contexts with conventional components to highlight device functionality. The key focus is the fabrication of an SMU with a graphene FET acting as the target device-under-test (DUT) to demonstrate the SMU’s performance.

## 2. Materials and Methods

In the following sections, SMU refers to the source measure unit that we developed and present in this paper, datalogger is used as a general term for any interfacing or data acquisition hardware that allows raw analog and digital data input and output under computer control, and probe station refers to a micro-positioning and micro-contacting device used for semiconductor die testing.

### 2.1. Probe Station

**Figure 2:**
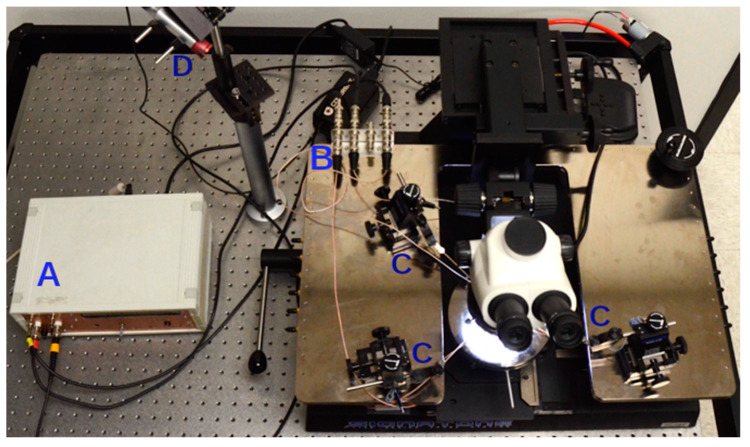
Photo of the semiconductor probe station connected to the source measure unit (SMU) hardware (**A**) presented in this paper. The probe station offers BNC terminals (**B**) for all three contact micro-positioners (**C**). A red laser (**D**) is mounted on a post and allows us to illuminate the device under test with varying light flux levels.

The key measurement apparatus to be integrated with our SMU is a probe station that allows for contact with micron-sized electrode pads on the target device. The Signatone S-1160 Series 1160 Manual Probe Station (Signatone Corp. Gilroy, CA, USA) [19] was used in this study. The probe station is designed for accurate contacting of semiconductor chips to enable analytical testing of the desired I-V curve. Die positioning and contacting are achieved by means of a vertically adjustable conductive center chuck, three micro-positioners with rectilinear X-Y-Z motion capable of probing down to one-micron pads, and tungsten needle tips to establish actual contact with the exposed pads. A low-power microscope with light-emitting diode (LED) illumination and a magnification range from 7.5× to 50× is mounted above the probe chuck and connected to the probe station body through a triaxial connector panel. As shown in Figure 2, the S-1160 probe station connects to our SMU acquisition electronics via three BNC connectors. These are connected with standard RG-58 BNC cables to our hardware. By using standard BNC connectors, our SMU design allows the sensor semiconductors to be probed both at the die level and at the chip level.

### 2.2. Source Measure Unit (SMU)

#### 2.2.1. Design Criteria

The first step towards the design of the SMU was determining current and voltage ranges to be sourced and measured for the graphene-channel FET. We also aimed to give the device enough flexibility to be used on different types of FET sensors. To achieve a high level of flexibility, we decided on a symmetrical driver capable of applying both negative and positive polarity, which allows us to measure the characteristic curves of, for example, n- and p-channel FETs and NPN and PNP transistors, and to allow a flexible choice of gate and drain voltage in FET sensors. Since one electrode is always the reference electrode (referred to as ground), three probes require two active and independent drivers. Both outputs, together with the reference ground, also comprise the three electrodes of a three-point measurement station. Whereas discrete transistors conduct comparatively high currents (several amperes for power transistors), the drain current for sensor FETs is typically in the order of several μA. We therefore decided to limit the voltage range from −10 V to +10 V and the current range from −100 μA to +100 μA. The range limits hold for both the active output and the measurement range. The voltage range also allows us to use conventional, off-the-shelf, symmetrical linear power supplies. The choice of a linear supply was made to avoid the switching noise typically caused by switch-mode power supplies, which is difficult to filter from the power rails and lowers the signal-to-noise ratio of the measurement signals. We also provided digitally controlled, selectable gain for each probe channel to better use the dynamic range of the digital-to-analog converters (DACs) and analog-to-digital converters (ADCs). Although it is theoretically straightforward to decrease digitization noise by employing higher bit-order ADCs and DACs (for example, 16 bits versus 12 bits), the least significant bits are dominated by noise. With adjustable gain, a lower-noise 12-bit converter can be employed with the same overall dynamic range.

#### 2.2.2. Device Overview

**Figure 3:**
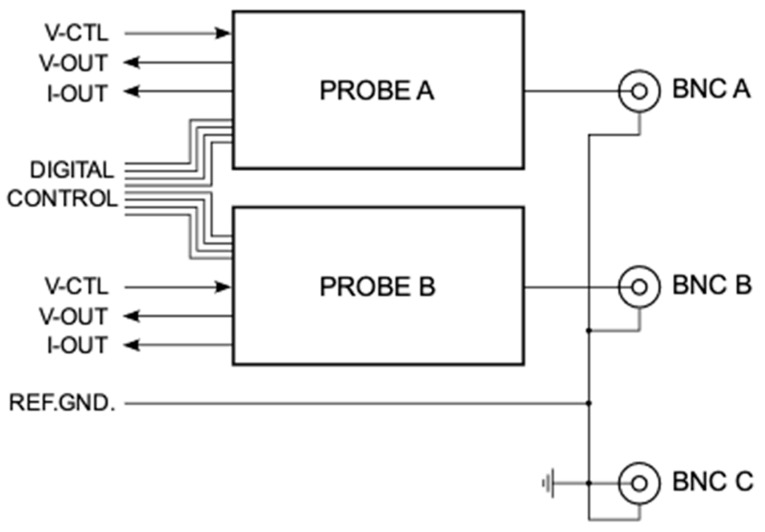
Overview of the SMU functional groups. Two identical active channels (A and B) serve either as a controlled-voltage or -current source with respect to the reference ground and Probe C. Each channel’s actual voltage and current are sensed and provided to the datalogger.

The presented device is described in detail in this section. The hardware of the SMU device was designed to be used in conjunction with general-purpose datalogger hardware, which is readily available from various manufacturers. Our lab is using a custom datalogger that is equipped with the AD7998 analog-to-digital converter and the AD5694 digital-to-analog converter (Analog Devices, Inc., Wilmington, MA, USA), both of which have a 12-bit resolution, unipolar 0 to 2.5 V input and output, and an I2C digital interface in common. However, any combined analog–digital interface system, such as National Instrument’s DAQ cards, is suitable as a datalogger and thus an acquisition back-end. A schematic block diagram of the SMU hardware and its interface signals is shown in Figure 3.

Two active channels A and B can be configured either as controlled-voltage or controlled-current sources, and their nominal output is determined by the control voltage V-CTL (Figure 3). Actual voltage and current are sensed at the probe output and provided as analog signals (V-OUT and I-OUT) that can be recorded by the datalogger. In a typical configuration, a metal–oxide–semiconductor FET (MOSFET) source would be connected to BNC connector C, its drain to connector A, and the gate to connector B. The control voltage for Probe A is then swept repeatedly with varying voltage levels for the gate at connector B. The ability to configure a channel as a controlled-current source was provided to extend the application range to non-FET semiconductors, including bipolar junction transistors, diodes, and even more exotic devices, such as unijunction transistors. Moreover, sensor FETs often use very low $V_{DS}$, often in the upper mV range. In such a case, it is more accurate to impose a constant current and measure the voltage needed to drive this current, which is a feature that the present device provides.

**Figure 4:**
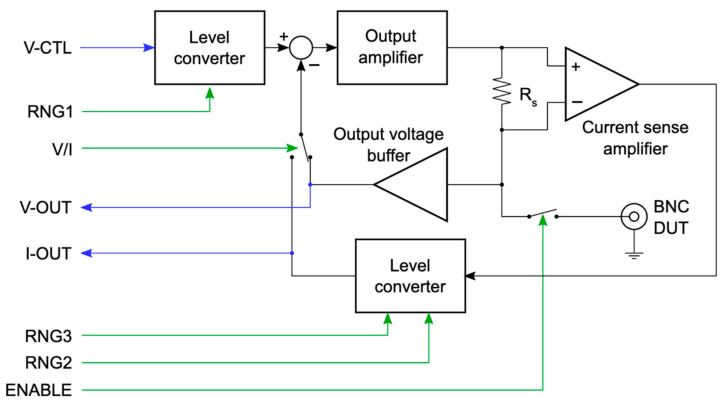
Detailed overview schematic of one of the two identical active channels. At the center is the output amplifier, which is embedded in a feedback loop to either provide a controlled current (I-mode) or a controlled voltage (V-mode). V- or I-mode is selected with an electronic switch. The current is sensed with the resistor R_s_, and the proportional voltage is presented at the interface, as is the actual probe voltage. Additional digital signals allow the selection of the current or voltage range, and a physical relay is provided to separate the DUT from the circuit until it has been fully configured.

A detailed functional block diagram for one of the two identical active probe channels is shown in Figure 4. At the center of each active probe channel is an amplifier that drives the load at the probe terminal. It is configured as a feedback amplifier that follows its input voltage either with the probe output voltage or with the voltage drop across a current sensing resistor R_s_, which is proportional to the current, and which is sensed with an instrumentation amplifier. Analog electronic switches, each controlled with a digital signal, allow us to select voltage- or current-controlled mode, introduce different gain factors that determine the measurement range, and make full use of the dynamic range offered by any SMU–datalogger combination.

The interface of the SMU and the datalogger in Figure 4 is labeled with blue arrows for analog signals and green for digital signals. The interface is straightforward to connect to a wide range of dataloggers or analog interface cards, and to further increase compatibility, the analog signals are level-shifted to a range from 0 to 2.5 V, which can be provided by single-supply devices. The full circuit schematics are provided in the Supplementary Materials.

#### 2.2.3. Modes

To make full use of a datalogger’s dynamic range, digital inputs allow us to select one of several sensitivity ranges, which were implemented as part of the level shifter circuitry. For example, a voltage range of ±1.25 V would be mapped to the ADC range of 0 to 5 V with a gain of 2 and an offset of 2.5 V. To measure the typically very low drain current in graphene-channel FETs, our device is capable of delivering and measuring only up to ±100 μA. The key factor in this design limitation is the sense resistor Rs with 2430 Ω. The resulting high impedance also acts as a protective factor for the attached device under test since it limits the maximum current to 12 V/2430 Ω = 4.9 mA under worst-case circumstances. An overview of the modes and measurement ranges is provided in Table 2. It is noteworthy that the limitation for the current range can be modified in a straightforward way: we placed the current sense resistor Rs in a socket. By replacing this resistor, the current range can be modified with minimal effort. For example, by using a resistor of 243 Ω, the current sensitivity ranges change to ±1 mA, ±250 μA, ±50 μA, and ±10 μA, respectively, because by decreasing the resistance by a factor of 10, the magnitude of the current modes increases by a factor of 10 owing to Ohm’s law. Conversely, if the resistor was changed to 24,300 Ω, then the current sensitivity would change in the opposite direction to ±10 μA, ±2.5 μA, ±500 nA, and ±100 nA, respectively. This setup allows for easy modification of the current range required for the graphene-channel FET. The upper limit is primarily imposed by the output driver, which is limited to approximately 20 mA. At the output driver’s limit, however, the device under test would be stressed with 200 mW, which causes considerable power dissipation in a graphene channel or comparable nanostructure. In fact, such power levels may already cause irreversible damage to the FET structure. For this reason, we did not implement any current-boosting amplifier stage beyond the driver output.

From the datalogger’s perspective, the modes are presented as 5-bit groups of digital signals (Table 2). A combination of these bits allows for different voltage and current ranges to be selected. For example, if the target graphene-channel FET gate is driven with ±10 V, the gate current, which should be zero, is monitored with the highest sensitivity (±1 µA). The sweep range would be set to ±10 V (+8), and the output range is also kept at ±10 V (+0 in Bit 4). The highest current sensitivity is selected with binary ‘11’ in Bits 1 and 2 (+6), and Bit 0 remains 0 for the voltage-controlled mode. The combination is binary 01110, decimal 14—this is the probe’s mode.

Conversely, if trying to measure the $I_{D}$–$V_{DS}$ curve with current control to obtain higher precision when the I-V curve becomes very steep while also limiting the current to 10 μA with $V_{DS}$ below 1 V, the current-controlled mode would be selected (+1), and the current sweep range would be ±20 μA (binary 01, +2). The voltage sweep range would be forced to ±1.25 V by the firmware, and Bit 3 would not be utilized (+0). The higher voltage sensitivity of ±1.25 V with Bit 4 (+16) is selected, and the combination yields the bit pattern 10011, decimal 19.

#### 2.2.4. Calibration

Component tolerances require per-channel calibration. Calibration equations linearly map voltage or current values to unitless digital values. If we denote the digital value as Z, an analog value (for example, output voltage $V_{a}$ of the active probe driver A) would be mapped through

(1) $$V_{a}=m_{a,M}\times Z+n_{a,M}$$

where $m_{a,M}$ is the slope for mode M (in this case, modes with voltage ranges of either ±10 V or ±1.25 V) and $n_{a,M}$ is the intercept. Component selection would allow us to calculate theoretical values for the mapping equations. For example, to map a 12-bit digital value Z to the voltage range of ±1.25 V, a slope of m = 0.61 mV/LSB and an intercept of n = −1.25 V would be expected. Due to component tolerances, deviations from the theoretical values can be expected, and a calibration regression provides more accurate values for the mapping slope and intercept. This type of calibration is usual for commercial precision instruments, which the manufacturer typically offers as a regular service.

For accuracy calibration, we used a Keysight 34461 A Digital Multimeter (Keysight, Santa Rosa, CA, USA) as a reference and connected it between the probe output and ground. To calibrate each of the SMU’s modes, different DAC values were applied. The associated ADC read-out for both current and voltage was recorded. Reference values for voltage and current were then read from the Keysight 34461 A digital multimeter and also recorded. For each mode, we collected 9 datapoints over the whole range of digital values (from 0 to 4095 in increments of 512) and performed linear regression to obtain the slope and intercept. For each active probe, the calibration data set therefore contained six data pairs of slope and intercept for the ADC (two voltage ranges and four current ranges) and six data pairs for the DAC.

Applying the digital data words and converting the digital read-out to actual physical voltage and current values is the task of the datalogger back-end, and the calibration data were programmed into the datalogger software. To apply a desired voltage $V_{a}$, Equation (2) is inverted to provide the desired digital DAC value Z:

(2) $$Z={m^{\prime }}_{a,M}\times V_{a}+{n^{\prime }}_{a,M}$$

**Figure 5:**
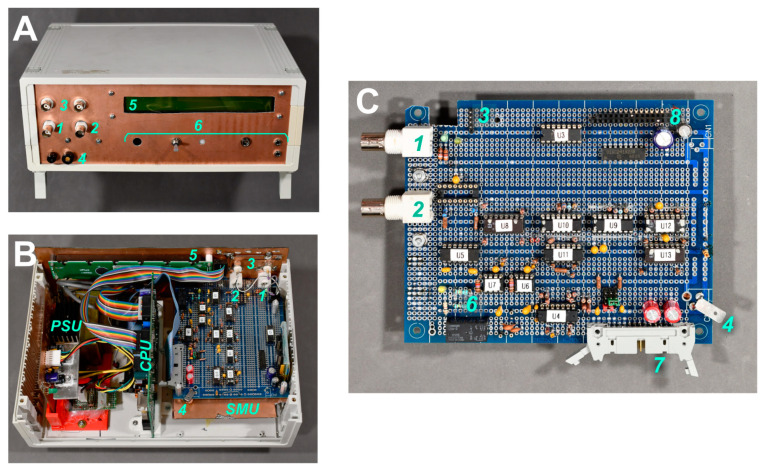
Photos of the SMU and datalogger component. (**A**) Exterior view showing the front panel, which is cut from a copper-clad printed circuit board (PCB) and grounded at the point labeled (4). The BNC connections for the active probes (1) and (2) and two identical ground probes (3) are visible; the ground probe is connected to its own analog ground. The front panel also includes an LCD (5) and additional functions (6) such as a trigger/abort switch, power output, and temperature sensor jacks. (**B**) Interior view of the entire device, which shows the SMU board, datalogger/CPU board, and the linear power supply (PSU) in context. The SMU board is shielded with copper-clad PCB. To make the SMU board visible, the upper shield, connected to (4) similar to the lower shield, was removed. (**C**) View of the SMU analog board: (1) and (2) are the active probe outputs, and (3) is the analog ground-referenced header for the passive probe connectors. The sensitive section of the amplifier circuits is limited to the ICs labeled U5 though U8 with the connectors (1) and (2). Also visible are the current gain resistors (6) held in sockets for easy replacement. A flat ribbon cable, connected at (7), carries the digital and low-impedance analog signals, and the header (8) can be used for an optional 16-bit port expander with a serial peripheral interface (SPI) with U1 and U2. The interface elements (7) and (8) allow the SMU to be connected to a wide range of data acquisition hardware with a minimum of four digital outputs, two analog outputs, and two analog inputs.

The datalogger then reads both analog outputs (Figure 5) through its ADC and converts the ADC digital values for voltage and current, $Z_{V}$ and $Z_{I}$, to physical quantities through

(3) $$V=m_{M}\times Z_{V}+n_{M}$$

and

(4) $$I=m_{M}\times Z_{I}+n_{M}$$

where again the index M stands for the mode. 

#### 2.2.5. Shielding

To minimize potential electromagnetic interference, shielding was added both internally and externally to block transient currents. The EMI shield was composed of double-sided copper-clad PCB material that was soldered to form a box enclosure for the SMU circuit board. The copper surfaces were electrically connected to the SMU ground terminal. Figure 5A shows the device layout and shielding additions. The circuit design itself also assisted in transient blockage through the separation of digital and analog signals using a specialized wiring scheme and focused on their positioning in the circuit, as seen in Figure 5B and in the Supplementary Materials. Our prototype was realized on a multi-bus prototyping board where connections beyond the bus traces were routed with 30 AWG insulated wire. No final PCB has been designed yet in order to confirm circuit functionality and allow flexibility in modifications; however, designing a fully custom PCB appears reasonable in the near future.

### 2.3. Software Interface

To automate acquisition of the V-I curves, the SMU needs to be programmed with a measurement loop that successively applies voltage or current values to the probe outputs and then takes a measurement for each probe’s actual voltage and current. Depending on the data acquisition system, the automated measurement loop can be programmed through some form of macro language (e.g., LabView) or a regular programming language, such as Python or C/C++. In our case, the high-level code was added to the datalogger’s GUI software written in C.

**Figure 6:**
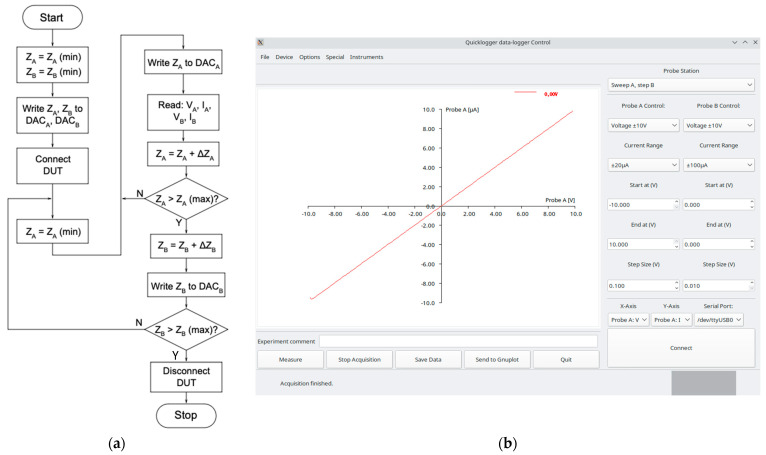
Software interface for source and measurement control of the SMU data collection: (**a**) flowchart; (**b**) GUI during measurement. The controls shown on the right side of the GUI act as spin-dials to trigger the flowchart from start to end. Current ranges from –100 μA to +100 μA and voltage ranges from –10 V to +10 V.

A flow diagram of the control software is shown in Figure 6a. As discussed with the linear regression used for calibration, Z represents the digital value that corresponds to an actual voltage or current in the SMU, with $Z_{A}$ representing the inner loop and $Z_{B}$ representing the outer loop. After values have been input, the program reads the actual voltage and current of both probes for confirmation as the setpoint values may not be reached due to saturation or component failure. The step size, ∆Z, is then applied to update the applied voltage or current, and the measurement repeated until the upper limit is reached. For Probe B, a new value is then applied, and the inner loop runs again for the new value at Probe B. For example, the inner loop can be set to apply a D-S voltage ($V_{DS}$) from zero to 10 V in 0.5 V increments, the acquisition is repeated with a gate voltage ($V_{GS}$) of 0, 1, 2, and 3 V for a total of four loop iterations, and 84 datapoints are acquired. After the last iteration, the DUT is disconnected from the SMU, and the process is complete. The associated GUI can be seen in Figure 6b.

### 2.4. Data Collection

The features of the SMU software and hardware allow for the key data collection required by the graphene-channel FET: $V_{DS}$ v $I_{D}$ curves with varying $V_{GS}$, and $V_{GS}$ v $I_{D}$ curves with constant $V_{DS}$ or, alternatively, $V_{GS}$ v $V_{DS}$ curves with constant $I_{D}$. As discussed, current and voltage can be set to various source and measurement range modes for greater accuracy. Probe A is set for continuous measurement while Probe B is set to collect a different curve per input at the specified step sizes and can be set to a constant if only one curve is desired. The data collected can be exported as a raw data file with 4 columns associated with Probe A voltage/current and Probe B voltage/current. Since all four values are kept in the measurement data set, the displayed data can be modified even after measurement.

For each test run, the modes for voltage and current are selected prior to inputting the start/end ranges and step size for the voltage or current supply. For $V_{GS}\;$v $I_{D}$, Probe A is connected to the gate with Probe B connected to the drain and GND connected to the source; Probe A is set to sweep across negative and positive voltage ranges for ambipolar curve visualization while Probe B is set to a constant voltage. For $V_{DS}$ v $I_{D}$, Probe A is set to sweep across varying voltage ranges used as constants during $V_{GS}$ v $I_{D}$ collection, while Probe B is set to step across different voltages to observe curve variation. Voltage is reflected on the y-axis with current on the x-axis, and leakage currents can be checked by selecting different probe I-V options to be reflected on the GUI measurement plane. Additional expected behavior for transfer and output characterization can be found in the Results and Discussion section below.

## 3. Results and Discussion

### 3.1. SMU Functionality Testing

**Figure 7:**
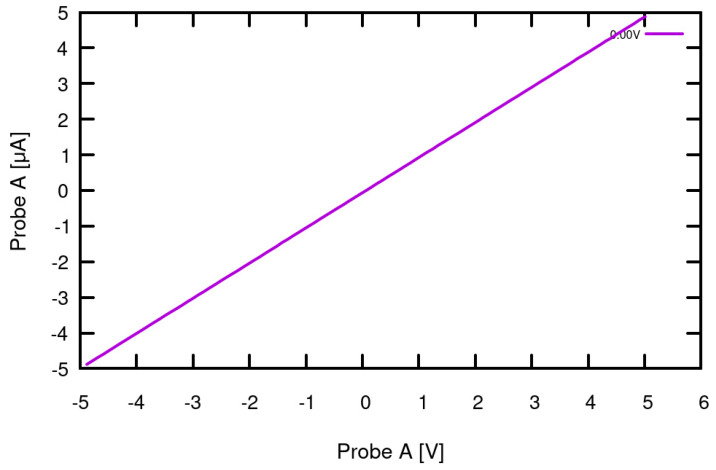
Results of 1 MΩ resistor test. The I-V curve shows the expected linear behavior of the resistor through the origin with a slope of 0.99 (spanning across ±5 μA—Current Mode 5) based on an input of ±5 V (Voltage Mode 8). The response validated the calibration and SMU functionality to allow for additional testing.

The basic function of acquiring I-V curves was first tested with standard electronic components with known responses. For semiconductors, the measured response was cross-referenced with its datasheet containing the identified curve behavior. The first device with which we demonstrate the SMU functionality is a carbon film resistor, 1 MΩ, ±5% tolerance (where the tolerance does not have an influence on the device accuracy, because it is the device under test and not part of the calibration). The resistor was connected to Probe A and Probe B in succession and for each current and voltage mode: Voltage Mode 8/16 + Current Mode 1/3/5/7 for a total of 16 tests. In accordance with Ohm’s law, the expected response for each resistor test was a linear curve through the origin with a slope of 1. As exemplified in Figure 7, when testing the 1 MΩ resistor across Probe A and GND for Mode 8 (voltage control = ±10 V) and Mode 5 (current range = ±5 µA), a linear curve from −5 V to 5 V and −5 µA to 5 µA through the origin with a slope of 0.99 was the result, confirming the datalogger’s functionality and validating calibration. The same data were collected for the remaining modes and Probe B.

**Figure 8:**
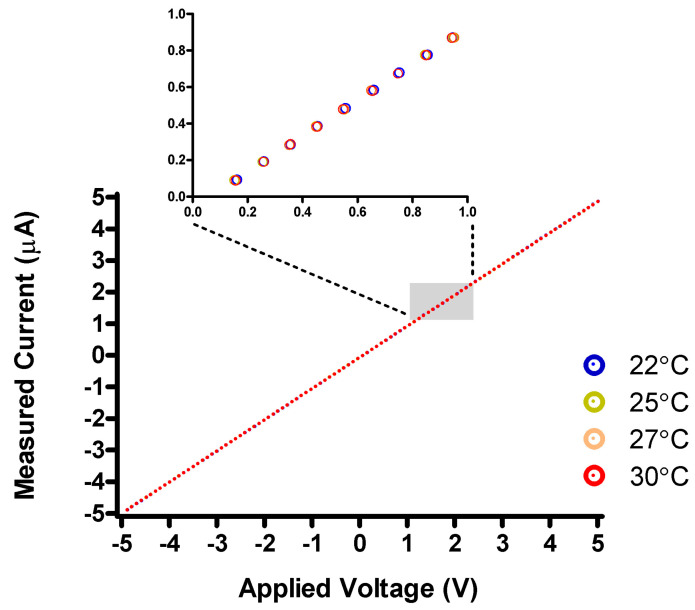
Test of temperature stability of the SMU. The SMU was connected to a 1 MOhm resistor and placed inside a cell culture incubator. For the experiment, CO_2_ was disabled. V-I measurement with the ±5 μA sensitivity range was performed at room temperature (22 °C) and repeated after one-hour equilibration at 25 °C, 27 °C, and 30 °C. The inset shows a magnified section (gray rectangle) to highlight the datapoints acquired at each temperature. Linear regression gives the slope (i.e., resistance in MΩ) as 0.9867 for 22 °C, 0.9852 for 25 °C, 0.9853 for 27 °C, and 0.9854 for 30 °C.

The same resistor was used for testing the robustness against temperature variations, and for accuracy estimation. Figure 8 shows the I-V curve of the test resistor, acquired after equilibration to 22 °C, 25 °C, 27 °C, and 30 °C inside an incubator. The device reported slopes from 0.9852 μA/V to 0.9867 μA/V, with data variability of 0.15% between the extremes. Despite the apparent robustness against thermal fluctuations within a limited range, it is conceivable that the SMU can be placed in a Peltier-controlled chamber to maintain a tightly regulated temperature. For the scope of our investigations into graphene FETs, we decided that such temperature control was not necessary.

The same 1 MΩ resistor was next used as a dummy load to compare the applied and measured voltages as reported by software to those reported by our reference multimeter, and the comparison is reported as root mean square error (RMSE, Equation (5)) in Table 3. At the same time, the signal-to-noise ratio (SNR) was calculated as the per-datapoint deviation from the best-fit straight line using the voltage and current values from Probe A. Our measurements indicated an SNR of about 63 dB or better, which makes it comparable to commercial devices on the market.

RMSE was tested by selecting voltage and current values across each range at each mode by software and measuring the actual voltage and current values with the reference Keysight 34461 A digital multimeter. We then calculated the error between the input and observed values across each probe. To present a metric for accuracy, we computed the RMSE (Equation (5)) for each range and mode:

(5) $$RMSE=\sqrt{\frac{1}{N}\sum_{k=1}^{N}\varepsilon_{k}^{2}}$$

where N is equal to the number of datapoints collected, i.e., 11 voltage/current values at each mode, and $\varepsilon_{k}$ represents the difference between the software-selected input data and actual observations (Y_input_ − Y_observed_) from the digital multimeter (here, Y may either be a voltage or a current). Only Mode 7 was not tested due to restrictions of the Keysight DMM.

**Figure 9:**
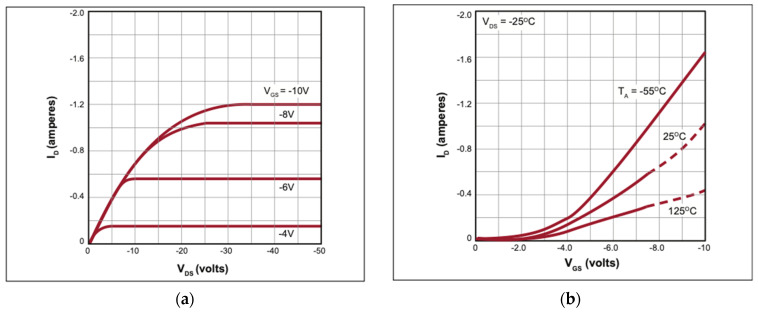
TP2450 p-channel MOSFET datasheet for output and transfer characteristics [20]: (**a**) output characteristics; (**b**) transfer characteristics. The I-V curves serve as the gold standard for the measurements collected in Figure 8 and Figure 9.

To further demonstrate the operation of the SMU, active components were introduced, and measurements compared to their expected output characteristics. The first device tested was a TP2540, a low-threshold p-channel enhancement-mode transistor with a vertical DMOS structure [20]. This device is known for its high current-carrying capacity; therefore, a 1 MΩ resistor was placed between the drain electrode and associated probe for data collection due to the SMU’s 100 µA maximum current range. The effect of the resistor can be seen on the output curves and serves as a demonstration of how the SMU handles compound devices, such as graphene FETs with their inherently large channel resistance. The datasheet for the TP2540 component was referenced to identify the general transfer characteristics of the device, as seen in Figure 9.

**Figure 10:**
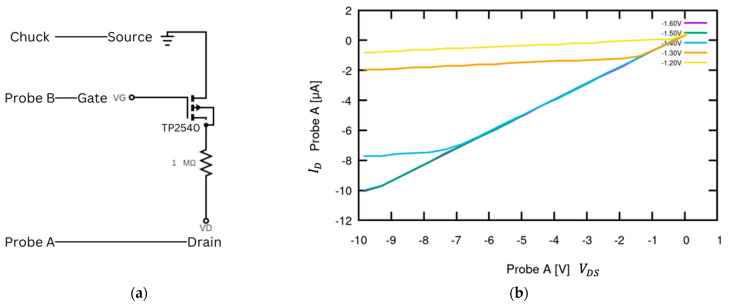
TP2540 P-channel MOSFET using SMU. (**a**) Schematic for $V_{DS}$ v $I_{D}$ curve. The source is connected to the SMU ground, Probe B is connected to the MOSFET gate, and Probe A is connected to the MOSFET drain. A 1 MΩ resistor was attached across the drain and Probe A to limit the current to the SMU max range. (**b**) $V_{DS}$ v $I_{D}$ curve. The collected curve can be compared to the datasheet in Figure 9.

The SMU was first used to confirm the $I_{D}$ v $V_{GS}\;$and $I_{D}$ v $V_{DS}$ curves for the TP2540. For the $I_{D}$ v $V_{DS}$ curves, the output characteristics were measured by looking at $I_{D}$ across $V_{DS}$ over various $V_{GS}$ values. The FET drain was connected in series with a resistor of 1 MΩ. The resistor acted as a current limiter and allowed us to vary *V*_DS_ over the full range of −10 V to 0 V without exceeding the SMU’s microampere measurement range. We focused on those values of *V*_GS_ where the MOSFET channel just opens, which happens between approximately −1.3 V and −1.4 V. For the measurement, the SMU’s ±20 μA current range (Mode 3) was selected, and the drain, connected through the resistor, was attached to Probe A. Probe B was attached to the gate and set to Mode 8 to observe $V_{GS}\;$at −1.6 V, −1.5 V, −1.4 V, −1.3 V, and −1.2 V, by inputting the voltage range as −1.6 V to −1.2 V with a step size of 0.1 V. These $V_{DS}$ values were selected in order to achieve the max current for testing device capability, and the current range of Mode 3 was again utilized. Probe A voltage ($V_{DS}$) is reported on the x-axis and Probe A current ($I_{D}$) is reported on the y-axis. As seen in Figure 10, the device was able to accurately measure the $V_{GS}\;$curves with the same overall behavior at lower current levels due to resistor impact.

**Figure 11:**
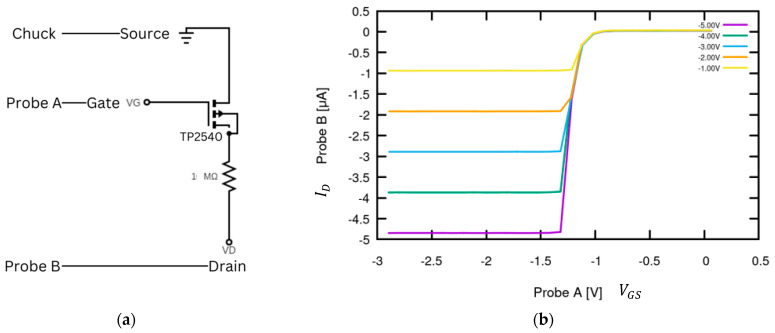
TP2540 P-channel MOSFET arrangement using SMU. (**a**) The source is connected to the SMU ground, Probe B is connected to the MOSFET drain, and Probe A is connected to the MOSFET gate. A 1 MΩ resistor was attached across the drain and Probe A to limit the current to the SMU max range. (**b**) $V_{GS}$ v $I_{D}$ curve. The collected curve was compared to the datasheet in Figure 7 to confirm device accuracy.

Following the output characterization of the TP2540, the transfer characteristics were collected for the $I_{D}$ v $V_{GS}\;$ curve, identified from the vertical cross-section of the $I_{D}$ v $V_{DS}$ plot to ensure measurements were taken across adequate voltage ranges. Probe A was attached to the gate at Mode 8 to test from $V_{GS}=$ −3 V to 0 V, as selected from the $I_{D}$ v $V_{DS}$ curves, and Mode 5 for an expected current range of ±5 µA. Probe B was attached to the 1 MΩ resistor for the discussed current suppression and then to the drain at Mode 8 to test across $V_{DS}=$−5 to −1 at a 1 V step size, with the current range set to Mode 5. Probe A was set for the x-axis voltage ($V_{GS}$), while Probe B was set for the y-axis current ($I_{D}$). As seen in Figure 11, the effect of the resistor is prominent, as seen by the steep L-shaped bend in the $V_{DS}$ curves. Without the presence of this additional resistance, the curves would continue to the maximum $I_{D}$ the device is capable of measuring at ±100 µA, as observed by the datasheet. This confirms the device’s ability to accurately measure the $V_{GS}$ v $I_{D}$ curve needed for future DUT data collection within programmed parameters. After testing both passive and active components with the SMU, the device’s functionality at low resolution was confirmed across voltage and current modes; therefore, the next step prior to graphene-channel FET data collection was integrating the SMU with a probe station to confirm testing integrity with an open-face BJT IC, as described in the section below.

### 3.2. SMU and Probe Station Integration Testing

**Figure 12:**
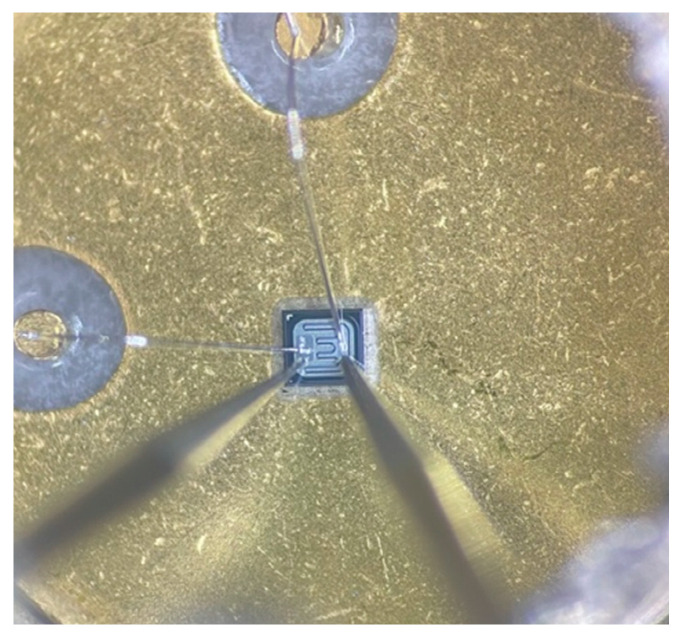
Acquisition of the electrical characteristics of an NPN transistor 2N3019. The measurement was performed to test the integration of the SMU with the probe station. For this purpose, the top of the TO-5 can was carefully removed to expose the transistor die. The base region (left probe needle) and emitter region (right probe needle) were directly contacted. The collector was connected through the conductive chuck.

The SMU was integrated with a 3-point probe station by using the BNC connection ports located on the probe station’s base. With a probe station, a semiconductor die is contacted with tungsten tips instead of the traditional contacts or pads of fully bonded semiconductors. The purpose of this step is to demonstrate the SMU in combination with the probe station to measure a semiconductor chip on the die level. A somewhat randomly selected sample transistor (2N3019) was prepared by removing the metal cover and thus exposing the transistor die. Figure 12 shows the microscope view of the probe station with the transistor die in the center and the two tungsten probe tips touching the metal areas of the die.

The left probe needle connects to the base area, and the right needle to the emitter area. The collector is connected to the case, as is common for this type of transistor. The case was coupled through the chuck of the probe station. The transistor curves were acquired in common-emitter configuration. Hence, the emitter was connected to the SMU’s ground, the collector to Probe A, and the base to Probe B. Analogous to the FET in Section 3.1, we acquired the curves of $I_{C}$ over $V_{CE}$ with varying base current values $I_{B}$. Unlike the FET, a bipolar junction transistor is a current-controlled device where the base current $I_{B}\;$influences the collector current $I_{C}$. Specifically, we expect $I_{C}$ to rise with $V_{CE\;}$up to a certain level (saturation level), above which $I_{C}$ cannot be further increased by increasing $V_{CE}$. The saturation level, however, increases with increasing $I_{B}$. The ratio of $I_{C}\;$ to $I_{B}$ (i.e., the transistor current gain) is typically between 100 and 300 for low collector currents.

**Figure 13:**
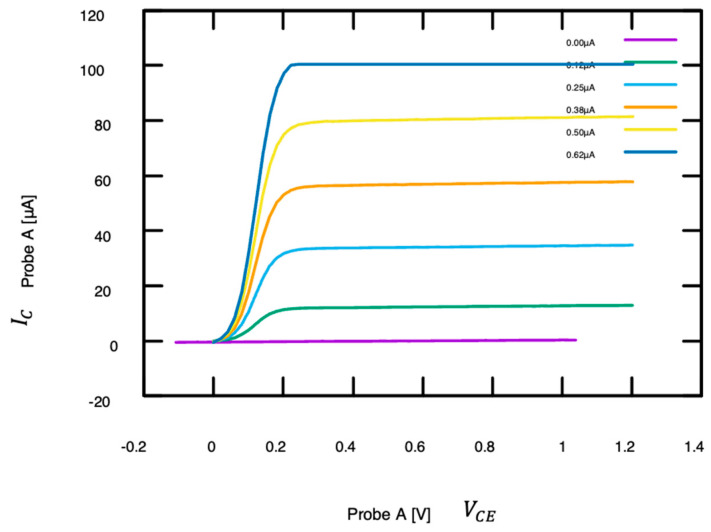
Family of *I*_C_ over *V*_CE_ curves for a medium-power NPN transistor (2N3019), probed at the die level. The curves were acquired at different base currents, and the linear region (*V*_CE_ < 0.2 V) can easily be distinguished from the forward active region (*V*_CE_ > 0.2 V). In the latter, *I*_C_ almost exclusively depends on *I*_B_ with the ratio *I*_C_/*I*_B_ ranging from approximately 100 to 160 in the covered range for *I*_B_.

The SMU was set up to acquire $I_{C\;}$in its maximum range up 100 μA with voltage steps for $V_{CE}\;$ of 20 mV from 0 V to 1.2 V (Probe A). The curve acquisition was repeated with the base current increased by 125 nA for each step (Probe B). We used the current-controlled mode with ±1 μA range, because even at the low current of 1 μA, the collector current would exceed the maximum of 100 μA. Once again, the high sensitivity (and therefore low limit) of the SMU’s current range is geared towards sensor FET and therefore not suitable for bipolar amplifier transistors. Figure 13 shows the family of $I_{C\;}$–$V_{CE}\;$ curves. Acquisition was stopped at $I_{B}$ = 0.625 μA because the saturation current for $I_{C\;}$exceeded 100 μA. Nevertheless, the linear region (up to approximately $V_{CE}\;$ = 0.2 V) and the forward active region (above $V_{CE}\;$ = 0.2 V) can clearly be seen. The large-signal current gain ranged from approximately 100 for $I_{B}\;$ = 125 nA to 160 for $I_{B}$ = 500 nA, which is consistent with the datasheet and with the nonlinear behavior of bipolar junction transistors at very low base currents.

### 3.3. Quality Testing: Parasitic Schottky Junction Formed by Oxide Breakdown

Another example that emerged from the practical application of the SMU is the possibility of identifying electrical or structural problems. Our graphene FET structure, as shown in Figure 1, builds on the substrate that also serves as the gate. Our FETs use gold electrodes as the source and drain, and gold pads are provided for the bond wires. We noticed occasional malfunctions of the graphene FET and explored these with the SMU. From Figure 1, we expected a gate current of zero but found that the current in Probe B rose steeply in one polarity. We hypothesized that the oxide layer had broken down under the mechanical power introduced by the bonding process, and that a gold-on-silicon Schottky junction had formed.

**Figure 14:**
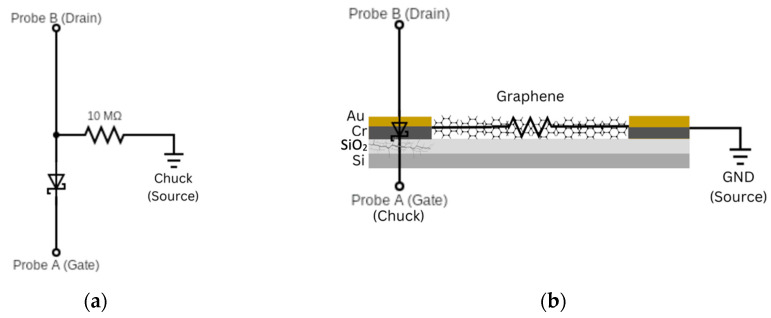
Schottky junction testing assembly. (**a**) Testing schematic: A Schottky diode was connected to Probe A in reverse polarity to mimic the metal-to-silicon junction that appears when the oxide breaks down, and Probe B was connected to the other side of the diode and a 10 MΩ resistor, which was also connected to GND. (**b**) Schematic of an actual graphene FET with the SiO_2_ layer damaged by excessive wire bonding force. The graphene-channel FET with a breached oxide layer on the drain electrode was compared to the testing setup for quality testing. Probe A was connected to the gate, Probe B to the drain, and GND to the source.

We first created a model of the hypothesized defect (Figure 14a) and performed measurements identical to those on the graphene FET. In the model, the electrode with the broken-down oxide was represented by a 1N5818 Schottky diode, and the graphene channel by a 10 MΩ resistor. The SMU would immediately reveal the diode with an I-V curve for the gate circuit, but our most common routine measurement is $I_{D}$ as a function of $V_{GS}$ with constant $V_{DS}$. Generally, the data set for $I_{G}\;$ is dropped in the process because it is expected to be zero.

**Figure 15:**
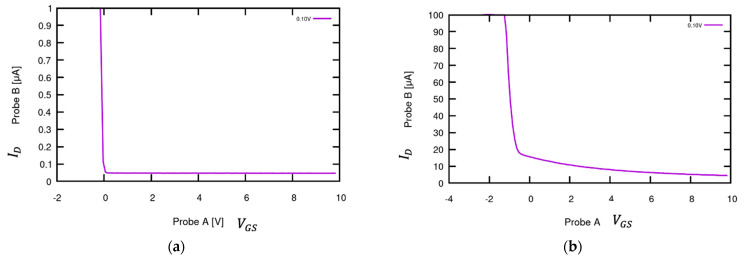
Schottky junction quality test results. (**a**) I-V curve of the model Schottky junction. The curve shows a steep increase in $I_{D}$ to reach the current level that the gate is driving at the negative $V_{GS}$ range on account of the Schottky diode. (**b**) I-V curve for the graphene-channel FET with breached oxide layer (Schottky junction). This behavior allows rapid assessment of faulty sensor chips.

Figure 15a shows the behavior of the model Schottky circuit. When the voltage of Probe A $V_{A}\;$ > 0, the diode is reverse-biased, and the current through Probe A is close to zero. When $V_{A}\;$ becomes negative, the diode begins to conduct and draw a large current from Probe B, which has the same direction as the voltage of Probe B and therefore appears positive. At the typical low currents, the voltage applied at Probe B approximately overcomes the diode forward voltage, and the transition is close to $V_{AB}\;$ = 0. Translated to the FET, a positive gate voltage would make the FET appear to operate normally, but when $V_{GS}$ turns negative, a rapid rise in the drain current can be observed (Figure 15b) [21]. We obtained further confirmation of this effect by exchanging source and drain, in which case no unusual drain current was observed, because the broken oxide was underneath the source and no longer influenced the drain current.

### 3.4. Demonstration of Measurements on Graphene-Channel FETs

**Figure 16:**
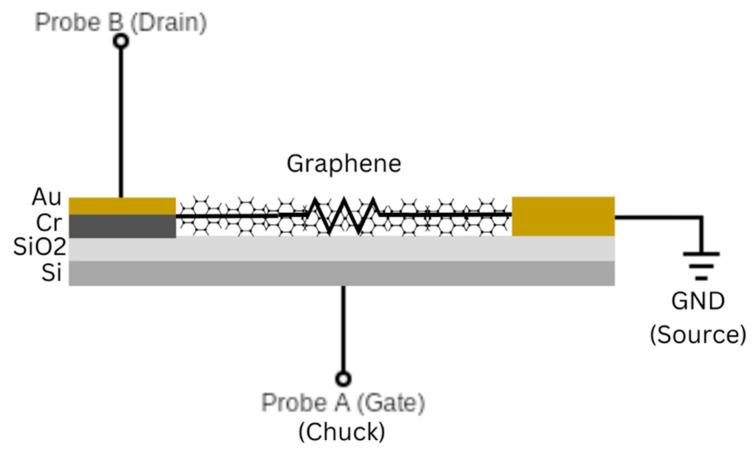
Graphene-channel FET schematic. As seen in Figure 1, the graphene is located across the source and drain electrodes for analyte detection. For the $V_{GS}$ v $I_{D}$ curve, the gate is connected to Probe A, the drain is connected to Probe B, and the source is connected to GND.

The graphene FET used as the target DUT was created using a p-doped silicon substrate with a thin oxide layer and gold source/drain electrodes on the sensor surface. The device has a back-gate structure with thin chromium and gold layers on top of the silicon serving as the gate and graphene across the source and drain electrodes serving as the gate channel. The G-FET schematic can be seen in Figure 16.

**Figure 17:**
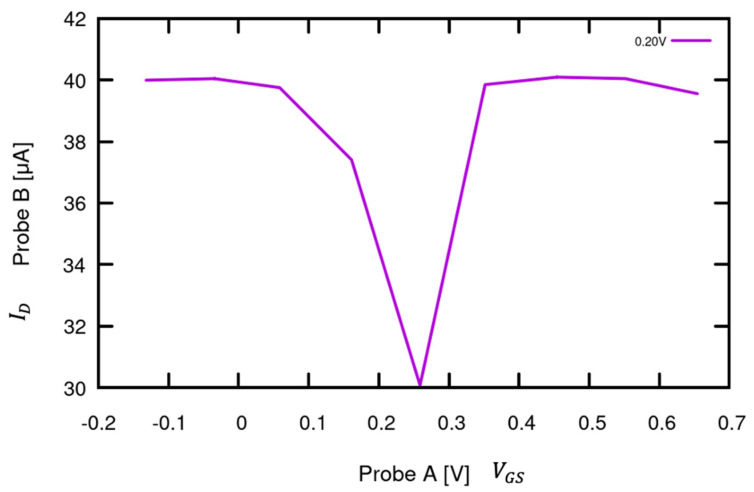
Graphene-channel FET: $I_{D}\;$ as a function of $V_{GS}$. Graphene allows conduction either through holes or through electrons. A negative gate voltage inhibits conduction through electrons, and $I_{D}\;$ is carried by the holes. Conversely, a positive gate voltage inhibits conduction through holes, and $I_{D}\;$ is carried by electrons. The point where the two carrier mechanisms balance out is known as the Dirac point and can be seen in this curve as the minimum $I_{D}\;$ of 30 μA when $V_{GS}$ = 0.25 V.

Similar to traditional FETs, the gate of the graphene-channel FET controls the flow of electrons or holes across the channel; all of the current flows across the graphene deposited between the drain and source (Figure 1). The graphene is exposed to the electrostatic field from the gate through a thin SiO_2_ layer. Graphene-channel FETs allow conduction through both electrons and holes, thus resulting in their ambipolar behavior in which the majority of carriers are holes during negative gate bias and electrons during positive gate bias. The two conduction bands meet at a neutrality point, known as the Dirac point, where the drain current, $I_{D}$, is lowest. The drain current should be zero; however, this point often shifts up due to doping, surface impurities, and contamination from the atmosphere [15]. The expected $V_{GS}\;$ curve is therefore a U-shape with the Dirac point located at the lowest $I_{D}$. In order to test the expected $V_{GS}$ v $I_{D}$ curve, the gate was connected to Probe A and voltage Mode 8 from −0.2 V to 0.7 V at current Mode 1 (100 µA) by placing the device on the probed chuck. The drain was connected to Probe B at Voltage Mode 16 with a constant 0.2 V and Current Mode 1. Probe A was set for the x-axis voltage ($V_{GS}$), while Probe B was set for the y-axis current ($I_{D}$). The measured response can be seen in Figure 17, the Dirac point is located at $V_{GS\;}=$ ~0.25 and $I_{D}=$ ~30 µA.

Photodetection is a key research area for improvement of FET optical sensing applications used here as an example. Improvement of the sensing mechanism for these devices would allow for optical communication, remote sensing, spectrum analysis, and biomedical imaging [22]. Researchers have investigated the transfer characteristic curves generated by photodetection on the G-FET surface, specifically that excitons are generated in graphene under various light conditions with response intensity influenced by symmetry in design and the wavelength of the light source. These excitons are separated into electrons and holes near the source and drain electrodes as a result of their corresponding electric fields [23]. For the design of the target DUT discussed here, the graphene was deposited closer in proximity to the source electrode within the gate channel, thus resulting in asymmetric behavior on the $V_{GS}\;$ v $I_{D}$ curve when compared to the control in Figure 17. This is due to the photovoltaic effect which generates increased photocurrents compared to a gate channel with a symmetric orientation [23] at the site of asymmetry. Since the graphene is deposited closer to the source, the negative gate bias generates a stronger drain current and an ambipolar response is no longer observed as the positive gate bias does not have an increased photocurrent. This leads to the conclusion that holes are the majority carriers present and reflected on the curve due to their relationship with the negative gate bias.

**Figure 18:**
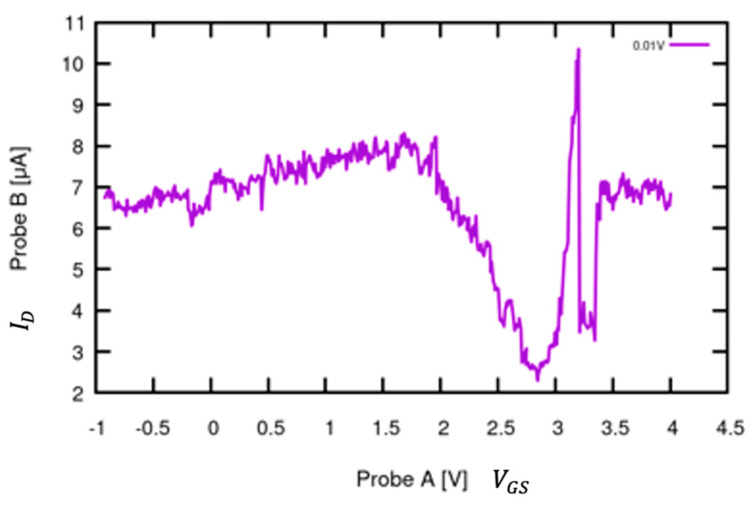
Graphene-channel FET $V_{GS\;}$ v $I_{D}$ control curve to juxtapose against laser response. The curve shown in Figure 17 was recollected using a different device in order to confirm functionality and prepare for laser illumination testing. The Dirac point shifts to the right and drops much lower compared to the first device tested ($V_{GS\;}=$ ~2.8 at $I_{D}=$ ~2.3 µA).

For testing, the same probe connections and test setup used for the control G-FET $V_{GS}$ v $I_{D}$ curve discussed above; however, a different device was used for repeatability purposes; therefore, a separate control $V_{GS}$ v $I_{D}$ curve was collected prior to laser illumination. Similar to the $V_{GS}$ v $I_{D}$ testing above, the gate was connected to Probe A at Voltage Mode 8 from −1 V to 4 V and Current Mode 1 (100 µA) by placing the device on the probed chuck. The drain was connected to Probe B at Voltage Mode 16 with a constant 0.01 V and Current Mode 1. Probe A was set for the x-axis voltage ($V_{GS}$), while Probe B was set for the y-axis current ($I_{D}$). The expected response can be seen in Figure 18; the Dirac point is located at $V_{GS\;}=$ ~2.8 at $I_{D}=$ ~2.3 µA. The curve reveals that different devices have different responses, required $V_{DS}$, and noise presence based on the orientation and concentration of graphene in the gate channel. The Dirac point for this device in comparison to the first decreases by over 28 µA and is shifted to the right by over 2.5 V.

**Figure 19:**
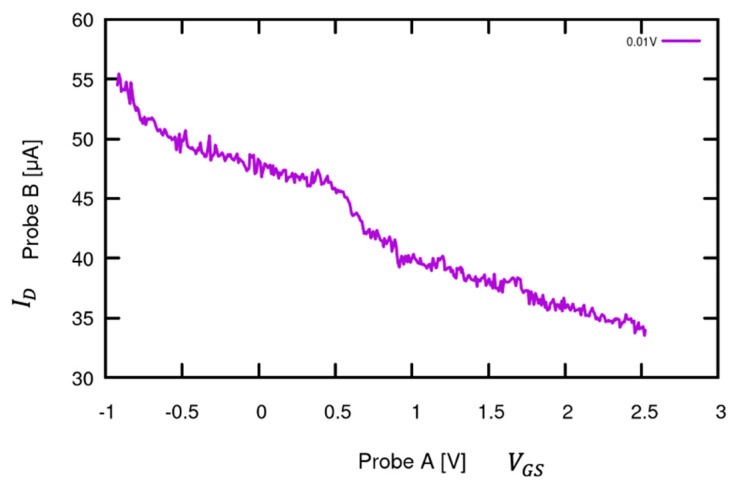
G-FET $V_{GS}$ v $I_{D}$ curve laser response. The same sensor used in Figure 18 was used with a laser light of wavelength 632 nm illuminating the graphene channel. The excitation resulted in the response shifting upwards by ~48 µA.

Once the control curve for this device was collected, a 632 nm, 0–10 mW solid-state laser (Coherent LabLaser series, Coherent Corp., Saxonburg, PA, USA) was used and pointed at the surface of the device angled at ~45 °. The gate was connected to Probe A at Voltage Mode 8 from −1 V to 2.5 V and Current Mode 1 (100 µA) by placing the device on the probed chuck. The drain was connected to Probe B at Voltage Mode 16 with a constant 0.01 V and Current Mode 1. Probe A was set for the x-axis voltage ($V_{GS}$), while Probe B was set for the y-axis current ($I_{D}$). The measured response can be seen in Figure 19; the increase in $I_{D}$ is extremely prominent in comparison to the control response, shifting upwards by ~48 µA for a negative gate bias, with the Dirac point no longer discernible.

When laser light photons are introduced to the graphene-channel FET, the ambipolarity of the $V_{GS}$ v $I_{D}$ curve reduces on account of several mechanisms. One significant factor is the reduction in carrier lifetime caused by photon-induced electron–hole pair generation. The continuous absorption of photons from the laser excites electrons from the valence to the conduction band, increasing the carrier density and thus diminishing the distinct Dirac point characteristic. The spatial location of photon impact can also play a role; due to the $V_{DS}$ gradient along the graphene channel, the influence of the gate field varies. Near the drain, the gate can be less positive than near the source due to the superposition of the positive gate field with the positive drain potential, leading to a varying electric field across the channel. This variation affects the distribution of photo-generated carriers and can cause the slope of the $V_{GS}$ v $I_{D}$ curve to greatly decrease on the side corresponding to positive drain current. Additionally, the interaction of laser photons with the graphene surface can induce the desorption of molecules adsorbed onto the graphene. These adsorbed molecules often act as charge impurities, influencing the local electrostatic environment and the position of the charge neutrality point. When these molecules desorb upon laser illumination, the local charge environment changes, shifting the Dirac point. This shift can result in an altered electric field distribution and carrier concentration, further contributing to the loss of the ambipolar response.

## 4. Conclusions

As nanomaterial-based FETs are growing in popularity due to their sensitivity and selective detection capabilities, graphene implementation has been largely investigated due to its tolerance for electron transfer through the entire FET structure, improving surface change response. Graphene-channel FETs utilize their sub-nanometer-thick sensing layer to measure electric field changes deeper within the gate channel, substantially improving sensitivity. Additionally, the ease of recognition molecule attachment to the graphene channel surface allows for target analyte selectivity. The presence of noise in the form of undesired analyte attachment in part due to the ultra-sensitivity of the device has presented challenges in graphene FET development, thus contributing to the need for a specialized measurement device that is tailored to its electrical characteristics. We demonstrated the SMU’s use in the specific example of the increase in $I_{D}$ due to laser illumination in graphene FETs, even though the potential applications go beyond this example.

We presented a source meter module that can interface with graphene-channel FETs on a chip level or, in combination with a probe station, at the die level. We have demonstrated the basic capabilities of the device, notably, the capability to measure responses in the sub-µA range, calibrated precision with root mean square errors near or below 1%, and a signal-to-noise ratio better than 60 dB. These performance metrics are comparable to commercial devices. The full schematics are published in the Supplementary Materials, and we believe that the circuit is straightforward to reproduce by researchers with moderate electronic skills. This robustness is the consequence of several design considerations and decisions that may not be immediately evident and are therefore listed here:

- Our main focus is on the analog component, but the device offers flexible interfacing options for mid-level data acquisition systems, DAQ cards, or dataloggers.
- The design includes digitally controlled options of per-probe voltage and current measurement, combined with the option to configure each probe as either controlled-voltage or controlled-current sources. All configurations are ambipolar. This flexibility allows us to measure and characterize semiconductors other than FETs, specifically those that require V-I curve measured under constant current conditions. Ambipolar probes also allow us to examine unknown sensors to identify their point of highest sensitivity with minimal assumptions.
- Digitally controlled switchable gain makes use of the dynamic range of low-cost ADCs and DACs with as few as 12 bits of resolution.
- Converters with higher bit resolution can be used to lower the quantization error, and an example configuration of the SMU with off-the-shelf PCI or USB data acquisition cards can be seen in the Supplementary Materials in Figure S2.
- The design is centered on the very low currents that are typical for semiconductor-based sensors, as demonstrated with graphene FETs in this manuscript.
- From an electronic design perspective, the analog part typically poses a greater challenge than the digital part. By focusing on the analog driver and measurement circuitry, the SMU is kept relatively straightforward with five analog ICs and approximately 35 passive components for one probe channel; low-impedance unipolar interface connections allow robust and uncritical adaptation to existing data acquisition infrastructure.
- The design is modular: The driver/measurement circuits for each probe are independent circuits that can be repeated if more than two active probes are needed.
- Although, at this point, no custom-printed circuit board was designed (to maintain design flexibility at the development stage), we expect that a suitably designed PCB can further improve the performance metrics. It is our plan to realize such a PCB, which will then be shared in electronic form similar to the schematics.

Numerous applications can be explored with the measurement device, such as for other gas, environmental, and biosensing uses. Moreover, the analog circuitry presented for the SMU is widely identical to the type of analog driver that is required to drive FET-based biosensors and for signal acquisition and conditioning. The SMU presented in this manuscript is therefore not only a precision tool to characterize FET-based biosensors, but it can also serve as a blueprint for the development of FET sensor drivers and read-out electronics.

## Figures and Tables

**Table 1:** Examples of commercial SMU units and comparison of technical specifications with our SMU.

Manufacturer	Model	Current Range	Voltage Range	Accuracy	Resolution (Current/Voltage)	Cost
Keithley [16]	2400 Standard Series	1 pA to 5 A	100 nV to 1100 V	0.0012%	1 pA/100 nV	USD 5280–USD 15,000
Keithley [16]	2650 Series	1 pA to 50 A	1 μV to 1100 V	0.08%	100 fA/100 nV	USD 27,400–USD 33,000
Keysight [17]	B2900 Series	10 fA to 10 A	100 nV to 210 V	0.015%	10 fA/100 nV	USD 5788–USD 13,814
Rohde & Schwarz [18]	NGU Series	200 pA to 10 A	5 μV to 20 V	0.025%	200 pA/5 μV	USD 5290–USD 8840
Our SMU	--	490 pA to 20 mA ^1^	1 μV to 10 V	0.02–1.09%	490 pA/1 μV	USD 400 ^2^

^1^ The upper range of our device is flexible and can be modified by swapping one resistor in the circuit, and the current range chosen is specialized to the DUT to avoid transient currents and can be easily adjusted. ^2^ USD 400 accounts for material costs. A complete list of materials is provided in the Supplementary Materials.

**Table 2:** Current- and voltage-controlled modes with built-in sensitivity ranges.

Combined Control Bits (Binary)	Bit 0: Control Mode	Bits 1, 2: Current Sensitivity and Range	Bit 3: Voltage Range	Bit 4: Voltage Sensitivity §	Resolution (per LSB), 12-Bit DAC and ADC
10xx0	0 (voltage)	†	0: ±10 V	(1)	4.88 mV
01xx0			1: ±1.25 V	(0)	0.61 mV
×1001	1 (current)	00: ±100 μA	‡	0: ±10 V or 1: ±1.25 V	48.8 nA
×1011		01: ±25 μA			12.2 nA
×1101		10: ±5 μA			2.44 nA
×1111		11: ±1 μA			0.49 nA

† Can be combined with any of the current ranges below. ‡ Must be logic high (±1.25 V) for current-controlled modes. § Must be opposite of Bit 3 in voltage-controlled modes. In current-controlled modes, either can be selected.

**Table 3:** RMSE for absolute accuracy calculations across each mode per probe.

Probe	Mode 1	Mode 3	Mode 5	Mode 7	Mode 8	Mode 16
A	1.09%	0.67%	0.43%	n/a	0.10%	0.01%
B	1.09%	0.67%	0.43%	n/a	0.10%	0.02%

## Data Availability

The original contributions presented in the study are included in the article/Supplementary Materials; further inquiries can be directed to the corresponding author.

## Supplementary Materials

The following supporting information can be downloaded at: https://www.mdpi.com/article/10.3390/s24123841/s1, Table S1: Bill of materials with sources and approximate costs; Figure S1: Full circuit schematics; Figure S2: Interfacing examples of the SMU with two examples of commonly used data acquisition systems; Table S2: Function of the SPI interface bits.
