# Supplementary material for: Low-Cost Source Measure Unit (SMU) to Characterize Sensors Built on Graphene-Channel Field-Effect Transistors
Source: Sensors (Basel). 2024 Jun 14;24(12):3841. doi: 10.3390/s24123841 (PMC11207584; doi:10.3390/s24123841)
Supplement: Supplementary file 1 [file sensors-24-03841-s001.zip › sensors-3028006-supplementary.pdf]

Supplementary Materials

Table S1: Bill of Materials with Sources and Approximate Costs

Line Item	Component	Manufacturer	Description	#	$
1	12.7 kΩ Resistor	Vishay	Metal Film Resistor, Rated ¼ W, 1% Tolerance	4	$0.40 ($9.90 for 100)
2	49.9 kΩ Resistor	Vishay	Metal Film Resistor, Rated ¼ W, 1% Tolerance	4	$0.20 ($5.10 for 100)
3	35.7 kΩ Resistor	Vishay	Metal Film Resistor, Rated ¼ W, 1% Tolerance	2	$0.20 ($9.90 for 100)
4	30.1 kΩ Resistor	Vishay	Metal Film Resistor, Rated ¼ W, 1% Tolerance	11	$1.10 ($9.90 for 100)
5	15 kΩ Resistor	Vishay	Metal Film Resistor, Rated ¼ W, 1% Tolerance	1	$0.06 ($5.90 for 100)
6	7.5 kΩ Resistor	Vishay	Metal Film Resistor, Rated ¼ W, 1% Tolerance	1	$0.06 ($5.90 for 100)
7	2.43 kΩ Resistor	Vishay	Metal Film Resistor, Rated ¼ W, 1% Tolerance	2	$0.20 ($9.80 for 100)
8	61.9 kΩ Resistor	Vishay	Metal Film Resistor, Rated ¼ W, 1% Tolerance	2	$0.20 ($9.90 for 100)
9	5.11 kΩ Resistor	Vishay	Metal Film Resistor, Rated ¼ W, 1% Tolerance	2	$0.13 ($6.60 for 100)
10	2.21 kΩ Resistor	Vishay	Metal Film Resistor, Rated ¼ W, 1% Tolerance	2	$0.20 ($9.90 for 100)
11	27.4 kΩ Resistor	Vishay	Metal Film Resistor, Rated ¼ W, 1% Tolerance	2	$0.20 ($9.90 for 100)
12	43.2 kΩ Resistor	Vishay	Metal Film Resistor, Rated ¼ W, 1% Tolerance	2	$0.16 ($8.30 for 100)
13	5.1 kΩ Resistor	Vishay	Metal Film Resistor, Rated ¼ W, 1% Tolerance	2	$0.13 ($6.60 for 100)
14	4.15 kΩ Resistor	Vishay	Metal Film Resistor, Rated ¼ W, 1% Tolerance	2	$0.20 ($9.90 for 100)
15	24.3 kΩ Resistor	Vishay	Metal Film Resistor, Rated ¼ W, 1% Tolerance	2	$0.20 ($9.90 for 100)
16	10 kΩ Resistor	Vishay	Metal Film Resistor, Rated ¼ W, 1% Tolerance	4	$0.12 ($6.20 for 100)
17	240 Ω Resistor	Vishay	Metal Film Resistor, Rated ¼ W, 1% Tolerance	8	$0.10 ($4.90 for 100)
18	2.55 kΩ Resistor	Vishay	Metal Film Resistor, Rated ¼ W, 1% Tolerance	2	$0.20 ($9.90 for 100)
19	1.27 kΩ Resistor	Vishay	Metal Film Resistor, Rated ¼ W, 1% Tolerance	2	$0.16 ($8.30 for 100)
20	2.15 kΩ Resistor	Vishay	Metal Film Resistor, Rated ¼ W, 1% Tolerance	2	$0.12 ($5.90 for 100)
21	1.5 kΩ Resistor	Vishay	Metal Film Resistor, Rated ¼ W, 1% Tolerance	2	$0.12 ($5.90 for 100)
22	390 Ω Resistor	Vishay	Metal Film Resistor, Rated ¼ W, 1% Tolerance	2	$0.10 ($4.90 for 100)
23	1 kΩ Resistor	Vishay	Metal Film Resistor, Rated ¼ W, 1% Tolerance	1	$0.06 ($5.80 for 100)

24	4.7 kΩ Resistor	Vishay	Metal Film Resistor, Rated ¼ W, 1% Tolerance	3	$0.20 ($6.60 for 100)
25	0.1 uF Capacitor	Murata	Multilayer Ceramic Capacitor - Leaded	4	$0.26 ($6.40 for 100)
26	0.22 uF Capacitor	Murata	Multilayer Ceramic Capacitor - Leaded	4	$0.42 ($10.40 for 100)
27	6.8 uF Capacitor	Murata	Multilayer Ceramic Capacitor - Leaded	2	$0.20 ($10.10 for 100)
28	470 uF Capacitor	Vishay	Aluminum Electrolytic Capacitor - Radial Leaded	3	$4.56
29	1000 uF Capacitor	Vishay	Aluminum Electrolytic Capacitor - Radial Leaded	1	$3.86
30	4.7 uF Capacitor	Vishay	Aluminum Electrolytic Capacitor - Radial Leaded	2	$3.46
31	TL074	Texas Instruments	Quad Low-Noise J-FET Input Operational Amplifier	12	$1.75
32	AD7998	Analog Devices	Analog to Digital Converter, 8-channel, 12-bit	4	$23.04
33	ADG513	Analog Devices	Precision Quad-SPST Switch, Noninverting	4	$16.00
34	ADG409	Analog Devices	Multiplexer Switch IC, 4-channel, +/-15V	4	$38.84
35	AD620	Analog Devices	Instrumentation Amplifier, Gain 1-10000	2	$22.76
36	HI-1-5043	Harris / Texas Instruments	Analog Switch IC, 16-channel	6	$60.00
37	DG411	Analog Devices	Quad-SPST Analog Switch IC	1	$13.19
38	DG413	Analog Devices	Quad-SPST Analog Switch IC	1	$13.19
39	SN74HC595	Texas Instruments	8-Bit Shift Register with 3-State 8-Bit Output Register	2	$1.00 ($13.50 for 25)
40	SSA36	OnSemi	Schottky Barrier Rectifier	1	$0.37
41	ULN2003	Texas Instruments	Darlington Transistor Low Power Relay Driver	2	$1.14
42	LED	Jameco ValuePro	Light-emitting Diode Standard Diffuse	3	$0.20 ($6.55 for 100)
43	BNC Connector	Jameco ValuePro	PCB Mount, Right Angle	2	$4.38
44	BNC Connector	Jameco ValuePro	Panel Mount	2	$3.90
45	AD5694	Analog Devices	Digital to Analog Converter, Quad, 12-Bit	2	$24.75
46	Omron G5V-2	Omron	General Purpose Relay, 5 VDC, Non-Latching, 2A, 125 VAC/DC	1	$3.55
47	3-Pin Jumpers	Jameco ValuePro	From 36-Pin 0.1” Breakaway Header, Straight	1	$0.99
48	Protoboard	Jameco ValuePro	PT Series Prototyping Board with 5 Power Buses	1	$9.45
49	Rat Wire	Jameco ValuePro	Wire Wrap 100 Feet 30 AWG	6	$100
Total					$356.08

Figure S1: Full Circuit Schematics

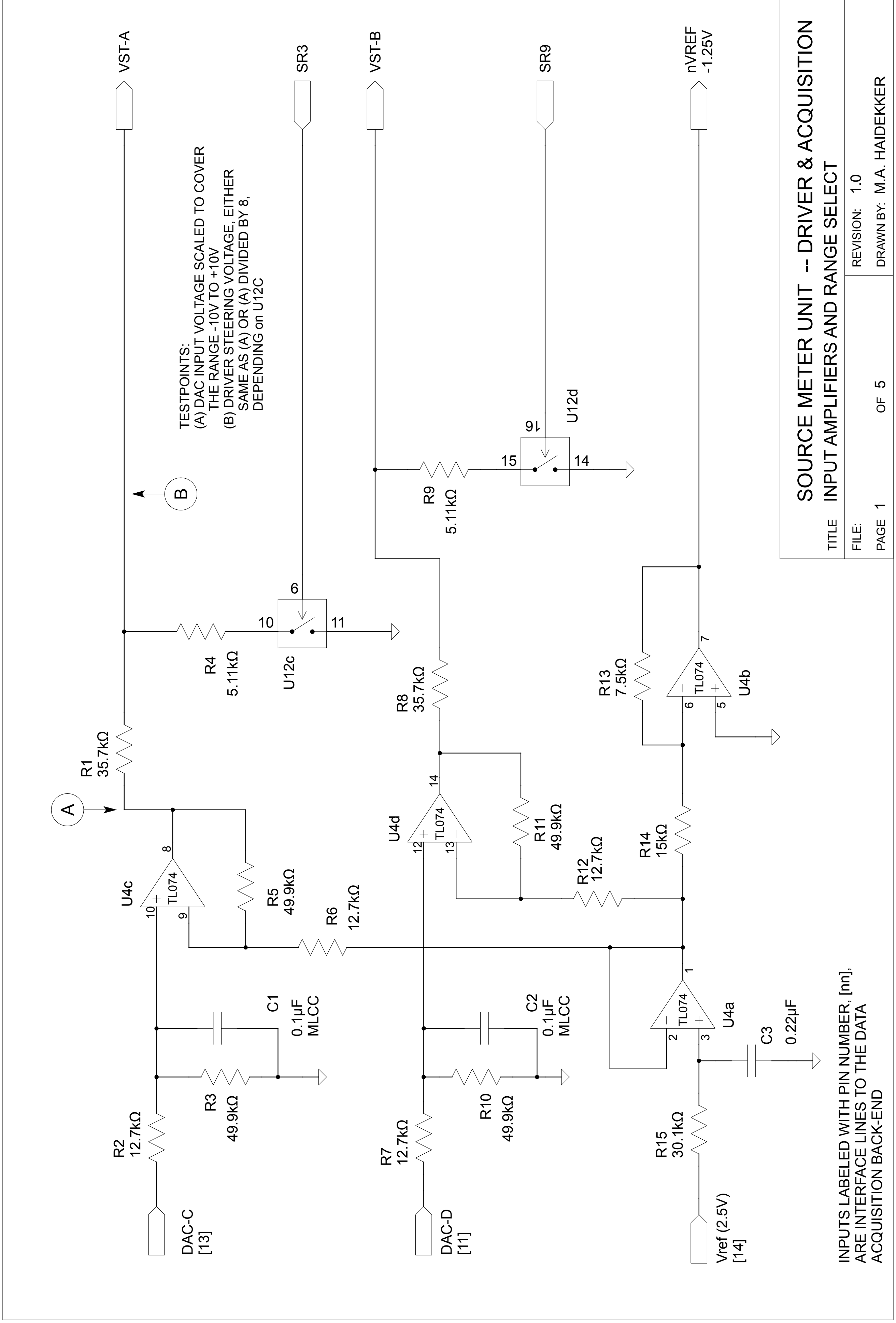

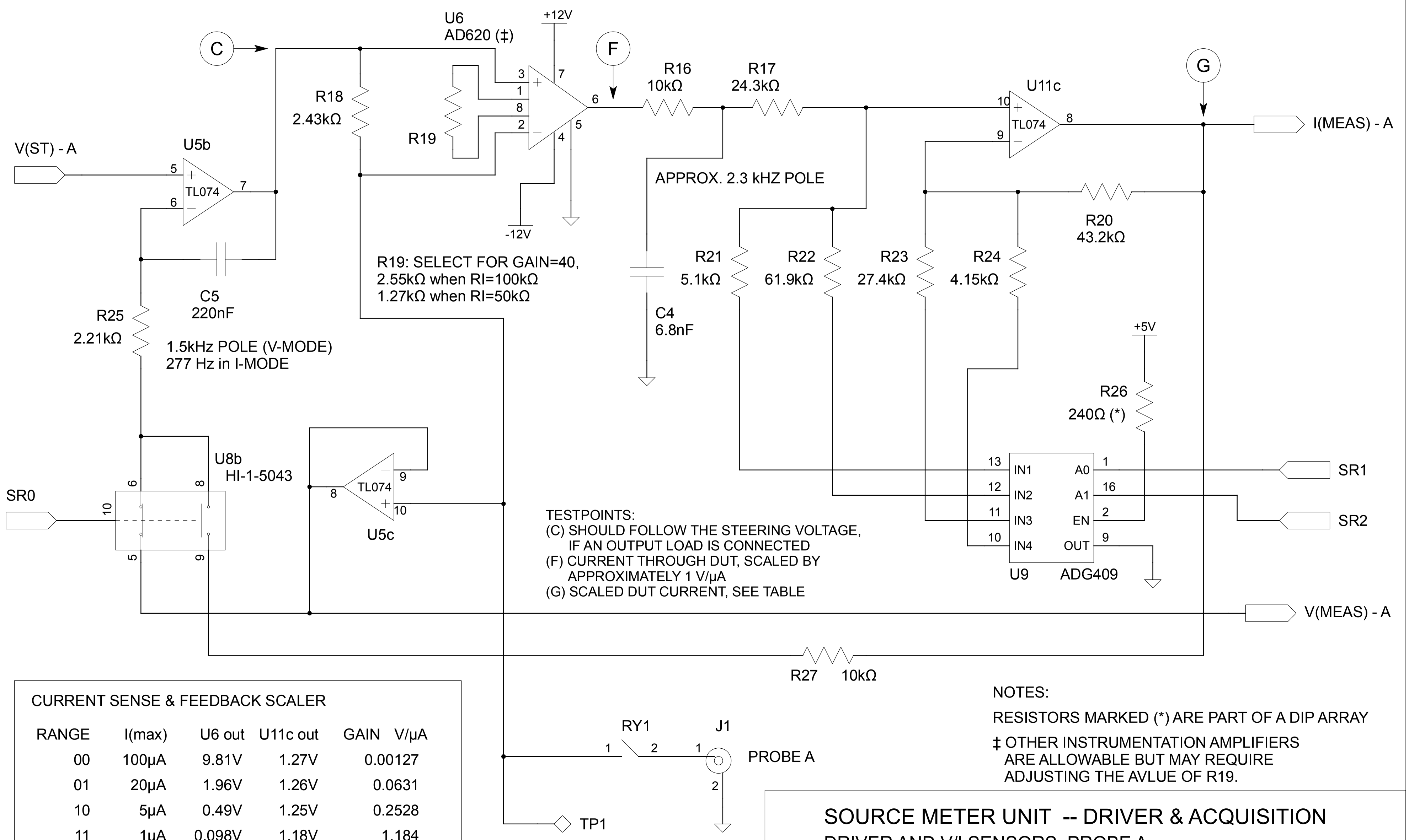

CURRENT SENSE & FEEDBACK SCALER

RANGE	I(max)	U6 out	U11c out	GAIN V/µA
00	100µA	9.81V	1.27V	0.00127
01	20µA	1.96V	1.26V	0.0631
10	5µA	0.49V	1.25V	0.2528
11	1µA	0.098V	1.18V	1.184

NOTES:

RESISTORS MARKED (*) ARE PART OF A DIP ARRAY

‡ OTHER INSTRUMENTATION AMPLIFIERS ARE ALLOWABLE BUT MAY REQUIRE ADJUSTING THE AVLUE OF R19.

TITLE SOURCE METER UNIT -- DRIVER & ACQUISITION<br>DRIVER AND V/I SENSORS, PROBE A	
FILE:	REVISION: 1.0
PAGE 2 OF 5	DRAWN BY: M.A. HAIDEKKER

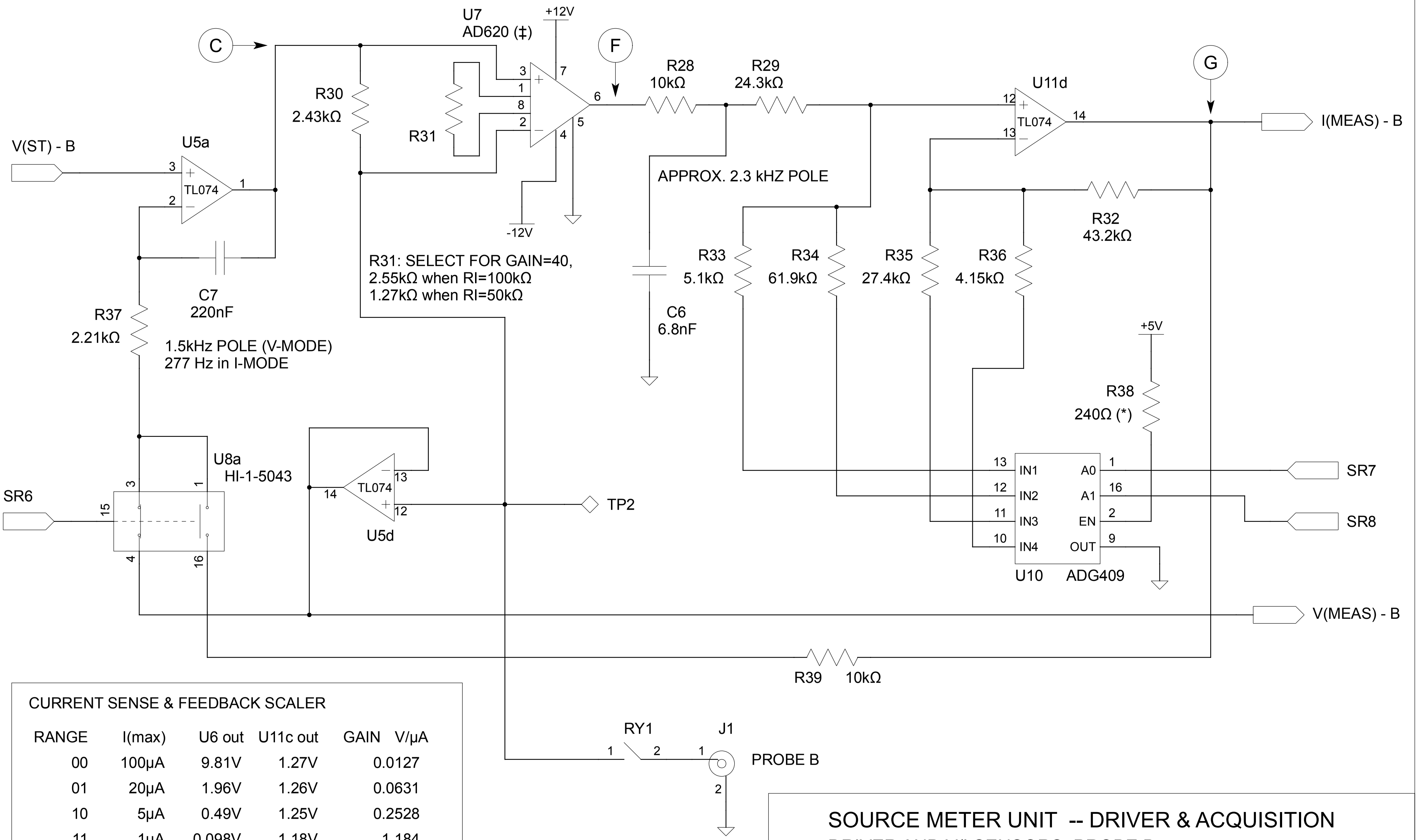

CURRENT SENSE & FEEDBACK SCALER

RANGE	I(max)	U6 out	U11c out	GAIN V/μA
00	100μA	9.81V	1.27V	0.0127
01	20μA	1.96V	1.26V	0.0631
10	5μA	0.49V	1.25V	0.2528
11	1μA	0.098V	1.18V	1.184

NOTEST AND TESTPOINTS:
SEE SHEET 2

TITLE SOURCE METER UNIT -- DRIVER & ACQUISITION<br>DRIVER AND V/I SENSORS, PROBE B	
FILE:	REVISION: 1.0
PAGE 3 OF 5	DRAWN BY: M.A. HAIDEKKER

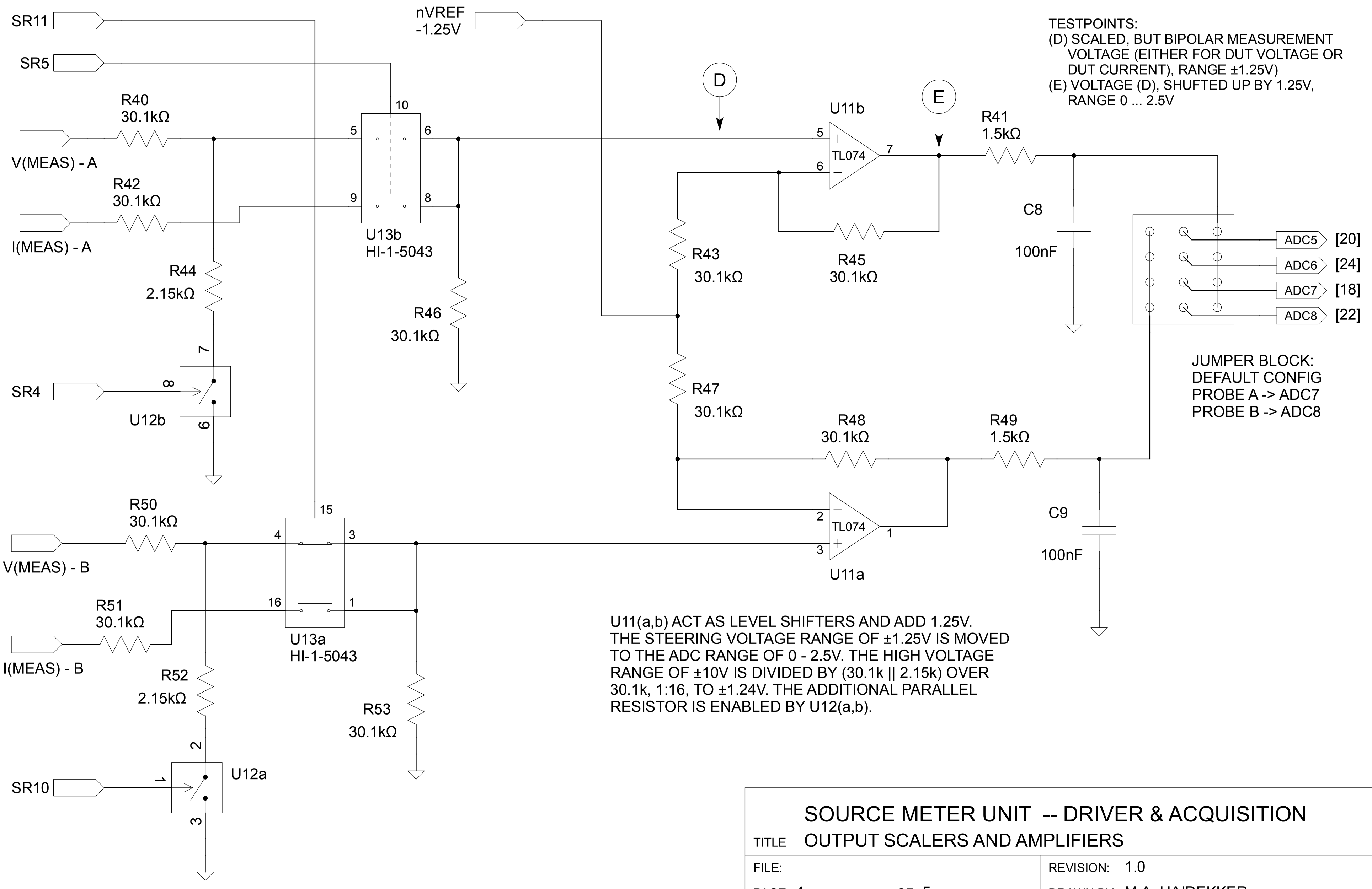

TESTPOINTS:
(D) SCALED, BUT BIPOLAR MEASUREMENT VOLTAGE (EITHER FOR DUT VOLTAGE OR DUT CURRENT), RANGE ±1.25V)
(E) VOLTAGE (D), SHUFTED UP BY 1.25V, RANGE 0 ... 2.5V

JUMPER BLOCK:
DEFAULT CONFIG
PROBE A -> ADC7
PROBE B -> ADC8

U11(a,b) ACT AS LEVEL SHIFTERS AND ADD 1.25V. THE STEERING VOLTAGE RANGE OF ±1.25V IS MOVED TO THE ADC RANGE OF 0 - 2.5V. THE HIGH VOLTAGE RANGE OF ±10V IS DIVIDED BY (30.1k || 2.15k) OVER 30.1k, 1:16, TO ±1.24V. THE ADDITIONAL PARALLEL RESISTOR IS ENABLED BY U12(a,b).

SOURCE METER UNIT -- DRIVER & ACQUISITION<br>TITLE OUTPUT SCALERS AND AMPLIFIERS	
FILE:	REVISION: 1.0
PAGE 4 OF 5	DRAWN BY: M.A. HAIDEKKER

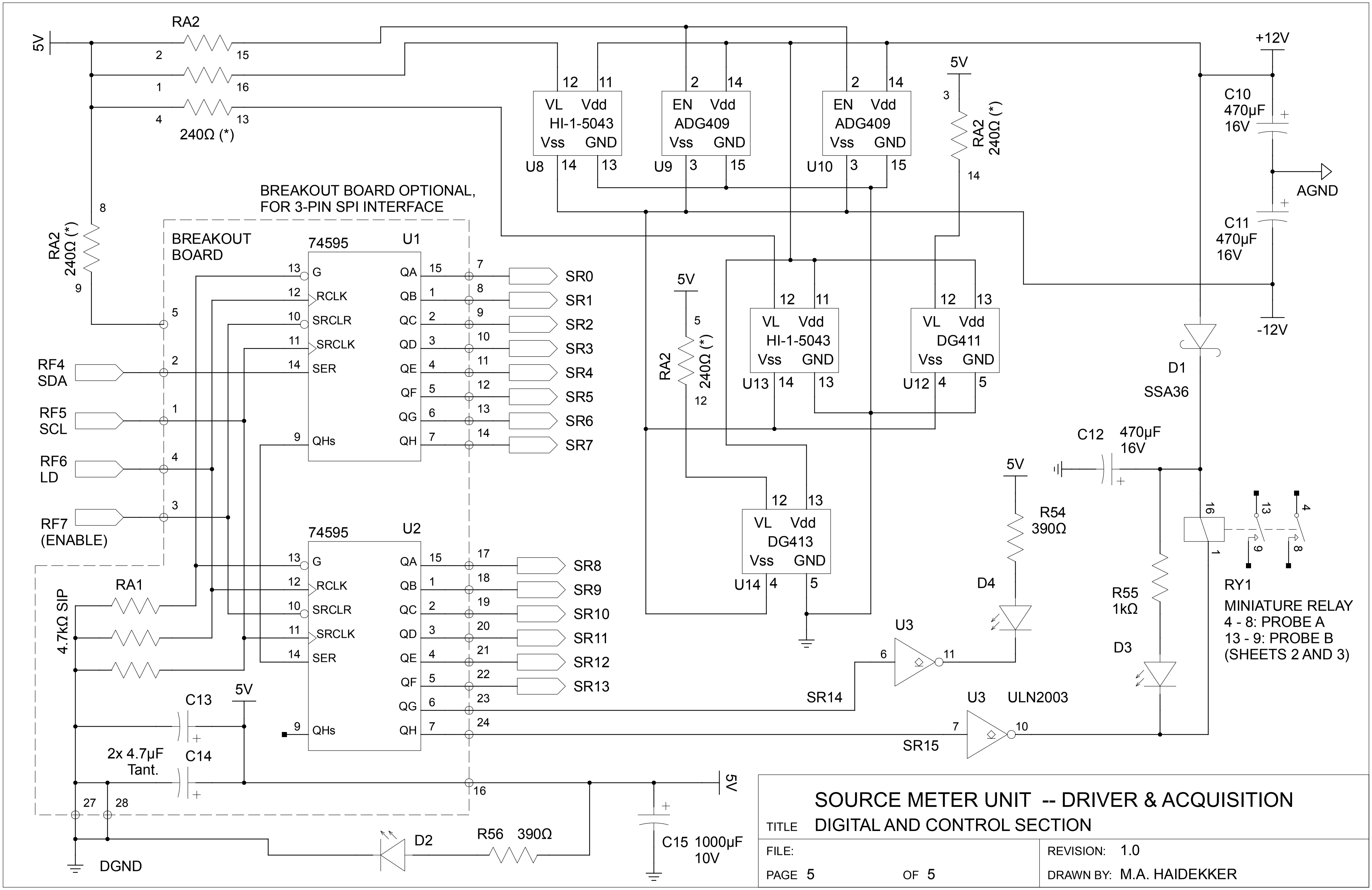

TITLE SOURCE METER UNIT -- DRIVER & ACQUISITION

DIGITAL AND CONTROL SECTION

FILE:

REVISION: 1.0

PAGE 5 OF 5

DRAWN BY: M.A. HAIDEKKER

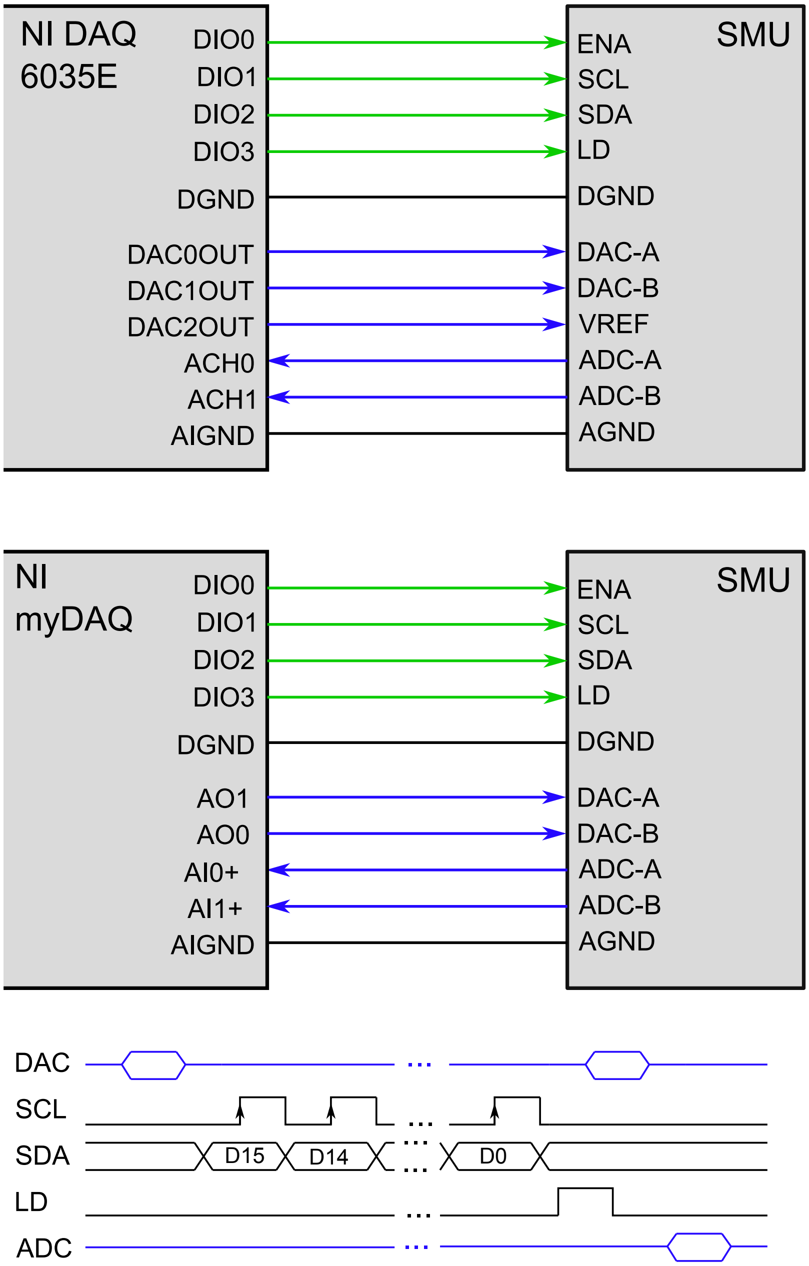

Figure S2: Interfacing examples of the SMU with two examples of commonly used data acquisition systems. (A) The first example is a National Instruments multi-channel analog/digital PCI card NI DAQ 6035E. (B) The second example is a low-end USB DAQ module, NI MyDAQ. As indicated in Figure 4, four analog signals are required. The digital signals can be provided by a four-wire SPI-type interface (Page 5 of Figure S1). The timing diagram for the SPI interface is provided in (C): The digital lines are shifted into the serial data line (SDA) LSB-first, and with a fixed 16-bit length. Each rising edge of the clock (SCL) samples the SDA input. Once all 16 bits are transferred, LD must be pulsed high for a few microseconds to propagate the input register to the outputs. The analog levels from the DAC can be updated both before or after the serial data transfer, but the analog acquisition (ADC) must take place after the digital transfer and the application of the DAC levels. Notably, the loop (Figure 6a) can be executed after the modes have been set once through the SPI interface, and merely updates of the DAC are needed. For an explanation of the 16 bits, see Table S2.

Table S2: Function of the SPI Interface Bits (see also Table 2)

Bit	Label in Schematics	Function
0 (LSB)	SR0	Probe A control mode (0: controlled voltage,<br>1: controlled current)
1, 2	SR1, SR2	Probe A, two-bit current range select<br>(00: ±100µA; 01: ±25µA; 10: ±5µA; 11: ±1µA)
3	SR3	Probe A input voltage scaler (0: ±10V; 1: ±1.25V)
4	SR4	Probe A output voltage scaler (0: ±1.25V; 1: ±10V)
5	SR5	Probe A measurement output (0: voltage, 1: current)
6	SR6	Probe B control mode (0: controlled voltage,<br>1: controlled current)
7, 8	SR7, SR8	Probe B, two-bit current range select<br>(00: ±100µA; 01: ±25µA; 10: ±5µA; 11: ±1µA)
9	SR9	Probe B input voltage scaler (0: ±10V; 1: ±1.25V)
10	SR10	Probe B output voltage scaler (0: ±1.25V; 1: ±10V)
11	SR11	Probe B measurement output (0: voltage, 1: current)
12		Not used, reserved for future extensions
13		Not used, reserved for future extensions
14	SR14	Signal LED, can be used to diagnose the interface
15	SR15	Output relay (0: Disconnect probe outputs from circuit, 1: Connect probe output to circuit)
